# Ciliary Functional Analysis in Chronic Rhinosinusitis with Polyps after Multimodal Intervention: Oral Corticosteroid, Functional Endoscopic Sinus Surgery, and Omalizumab Injection

**DOI:** 10.1155/2024/5559001

**Published:** 2024-05-14

**Authors:** Lionel Benchimol, Olivier Bouchain, Noemie Bricmont, Romane Bonhiver, Celine Kempeneers, Philippe Lefebvre, Anne-Lise Poirrier

**Affiliations:** ^1^Centre Hospitalier Universitaire de Liège, Avenue de l'Hôpital 1, Liège, Belgium; ^2^Pneumology Laboratory, I3 Group, GIGA Research Center, University of Liège, Liège 4000, Belgium; ^3^Division of Respirology, Department of Pediatrics, University Hospital Liège, Liège 4000, Belgium

## Abstract

In her late 50 s, a woman with a medical history of endoscopic sinus surgery for chronic rhinosinusitis with nasal polyps (CRSwNP) experienced a relapse of nasal polyps, significantly impacting her breathing and sense of smell. She underwent a multifaceted treatment approach, including oral corticosteroids, functional endoscopic sinus surgery, and omalizumab injections. Digital high-speed videomicroscopy (DHSV) revealed only partial improvement in ciliary beat pattern and ciliary beat frequency with oral corticosteroid treatment, while significant improvement in these ciliary parameters was observed with omalizumab injections. Furthermore, administration of omalizumab resulted in a decrease in her SNOT-22 (Sinonasal Outcome Test 22) score. Notably, this case report represents the first study investigating ciliary function using DHSV in a patient treated with omalizumab.

## 1. Introduction

Clearance and protection of the respiratory tract involve periciliary fluid and mucus production and drainage, as well as ciliated epithelium function. Disruption of mucociliary clearance may be caused by primary diseases such as cystic fibrosis or primary ciliary dyskinesia, or may be secondary to toxic exposure, infections, or chronic disease such as chronic rhinosinusitis [1]. In case of ciliary dyskinesia secondary to chronic rhinosinusitis, the ideal treatment would target the causative disease in order to restore the physiological function and the capacity to respond to future illness. Compromised cilia motility accompanies epithelial hyperplasia in individuals with nasal polyps (NP) [2]. This impairment in ciliary function is thought to likely contribute to persistent mucosal inflammation or infections, such as biofilm formation, commonly seen in people with chronic rhinosinusitis [2]. In addition to epithelial cell dysfunction, there is a prevalent type 2 inflammatory pattern in chronic rhinosinusitis with nasal polyps (CRSwNP) in Western countries, characterized by the expression of interleukins (ILs) IL-4, -5, and -13, as well as elevated IgE concentrations [3]. This trend was observed in 85% of patients with CRSwNP [3].

Chronic rhinosinusitis with nasal polyps (CRSwNP) is associated with elevated IgE production and eosinophilic inflammation [4]. Omalizumab, an anti-IgE antibody, has been shown to be effective in patients with both CRSwNP and coexisting asthma [4]. In fact, omalizumab has demonstrated its efficacy in dampening the type 2 inflammatory response in CRSwNP [4]. This reduction in inflammation may contribute to improved ciliary function by relieving the chronic irritation and inflammation [2].

Unfortunately, to date, no studies have specifically investigated the ciliary function both before and after administration of omalizumab treatment in a patient presenting with CRSwNP. Assessment of ciliary function is achievable through digital high-speed videomicroscopy (DHSV) [5–7]. DHSV facilitated the examination of ciliary beat frequency (CBF) and beat pattern (CBP) [8].

This case report investigates ciliary epithelium function throughout chronic rhinosinusitis management by corticosteroids, surgery, and the administration of subcutaneous omalizumab.

## 2. Case Presentation

A Caucasian female in her late 50s with a history of chronic rhinosinusitis with nasal polyps presented to the ENT outpatient clinic after an unsuccessful course of oral and local corticosteroid. She had a history of functional endoscopic sinus surgery for obstructive nasal polyps 5 years before. The ENT examination with nasal endoscopy showed grade 3 polyposis in both nasal cavities. The SNOT-22 score was 45/110. The blood eosinophils count was normal (120/mm^3^, 1.6%) and the total serum IgE was increased. At this time, the patient was listed to undergo functional sinus surgery and omalizumab injection afterwards. Pathological evaluation confirmed the eosinophilic nature of the polyps. Starting on the 5th month following surgery, a regimen of omalizumab treatment was administered monthly. At baseline, total IgE levels stood at 464kU/l. Clinical and endoscopic follow-ups were conducted up to 1 year post-surgery and 7 months post-omalizumab initiation.

In our case, four samples of ciliated epithelium were obtained from nasal brushing without local anaesthesia of the middle turbinate under endoscopic view, and ciliary function was analyzed using DHSV.

Nasal brushing samples were placed in 2 ml of medium 199 (Thermo Fisher, Waltham, MA, USA) containing an antibiotic solution (1% penicillin/streptomycin (Thermo Fisher, Waltham, MA, USA)) and an antifungal (1% amphotericin B (Thermo Fisher, Waltham, MA, USA)). Video sequences of ciliated beat edges were recorded using an inverted microscope with a 100x oil immersion interference contrast objective (Axio Vert.A1, Zeiss, Oberkochen, Germany) and a video camera at high speed (CrashCam Mini 1510, IDT Innovation in motion, Pasadena, CA, USA), at a frame rate of 500 hertz (Hz) and at a controlled temperature of 37°C. To record video sequences of cilia beating, 60 *µ*L of respiratory ciliated edges in medium 199 was placed under the microscope and heated to 37°C using a heated box (Ibidi, Gräfelfing, Germany) and a microscope lens heater (Tokai Hit, Fujinomiya, Japan), and the temperature was strictly controlled and stable at 37°C [9].

Solely the edges considered normal or having minor projections, measuring at least 50 *µ*m in length, were documented and utilized for ciliary functional analysis (CFA) [10]. Among these edges, only cilia free of mucus and displaying sideways beating profiles were examined. CFA was assessed based on a minimum of 3 high-quality edges meeting the specified criteria for each condition.

To perform manual assessment of ciliary beat frequency (CBF), the assessment involved identifying cilia or groups of cilia that beat in the sideways profile. The process included counting the number of frames needed to complete 5 beat cycles, which was then converted to CBF through a simple calculation [8]. A maximum of 10 CBF measurements were obtained from each ciliated beating edge. Ciliated edges that did not allow at least 4 CBF measurements to be performed were excluded from the analysis. If immobile cilia were present, a CBF of 0 Hz was recorded [9]. The average CBF for each sample was calculated for each treatment condition.

The specific movement of a cilium or group of cilia throughout a complete beating cycle was compared to the normal ciliary beating pattern (CBP) observed with DHSV [8, 11]. Each cilia or ciliary group evaluated was classified as having a distinct normal or abnormal CBP. The percentage of normal CBP in the sample was calculated for each condition [9].

The first sample was before functional endoscopic sinus surgery; the patient was under a treatment of oral corticosteroids. The second sample was three months after surgery, and the patient used only nasal corticosteroids and did not use it the day of sampling collection. The third sample was on the day of the third omalizumab injection, approximately 1 month after the first injection. Finally, the fourth sample was the day of the ninth omalizumab injection, approximately 3 months after the first injection. The results of the samples are presented in Table 1.

The patient showed a notable improvement in her condition, marked by a significant reduction of symptoms. Following treatment, the patient reported a remarkable improvement in her sense of smell, accompanied by improved sleep quality and a notable increase in her overall energy levels daily. Additionally, as a precautionary measure, the patient now undergoes regular ENT check-ups every three months, involving nasal endoscopy to monitor any signs of recurrence or polyp formation.

Patients with comorbid nasal polyps and asthma present a better reduction in asthma exacerbation rate, asthma control tests, rhinosinusitis outcome, and related quality of life under omalizumab compared to control patients [12]. However, the exact mechanism is still under investigation [13]. Our case report suggested that the mechanism may involve airway remodelling, including increase of cilia presence, allowing better local ciliary function [14]. Domingo Ribas et al. showed that baseline membrane thickness and intercellular spaces were reduced, and epithelial damage was improved [14]. Effective interaction between the mucus layer and coordinated ciliary beating resulted in better mucociliary clearance and symptom improvement.

## 3. Discussion

A remarkable observation in this case study is the similarity in ciliary function (CBF and CBP) between sample #1 (before FESS, with treatment of oral corticosteroids) and sample #4 (after three months of omalizumab injections). This similarity implies that treatment with oral corticosteroids may have played a crucial role in maintaining ciliary function by reducing inflammation even before the start of omalizumab treatment.

Sample #1 demonstrated a CBF of 16.78 ± 1.51 Hz, which remains consistent with sample #4 (16.03 ± 1.72 Hz). Similarly, the CBP in sample #1 was 95.65% and sample #4 had a CBP of 100%. This suggests that treatment with oral corticosteroids effectively preserved ciliary function, possibly by attenuating inflammation of the nasal mucosa. In fact, previous study demonstrated the direct influence of cytokines on ciliary function in the respiratory epithelium, contributing to impaired mucociliary clearance in a cell culture model of ciliated human respiratory epithelial cells [15]. Specifically, interleukin-4 (IL-4) and IL-13, which are associated with the T helper (TH) 2 cytokine profile, have been shown to decrease CBF, while IL-5 and l IL-9, also TH2 cytokines, lead to an increase in CBF [15]. However, another hypothesis suggests a potential link between ciliary dysfunction and chronic mucosal inflammation or infection observed in patients with CRSwNP [2]. In fact, nasal polyps can lead to abnormal cilia architecture, causing alterations in motile cilia and ciliogenesis-associated markers, which subsequently affect ciliary beat frequency (CBF) [16–18]. This abnormal architecture, characterized by untidy, overly dense, and lengthened cilia, has been observed in patients with nasal polyps both in vivo and in vitro [16, 17]. Furthermore, studies have demonstrated that the overexpression of ciliogenesis-associated markers in patients with nasal polyps is associated with abnormal cilia architecture, potentially contributing to the observed CBF slowdown [16, 18, 19].

The initial decrease in ciliary function observed in sample #2 three months after FESS may be attributed to the switch from oral to nasal-only corticosteroids. This transition could have temporarily impacted inflammatory control, leading to a transient reduction in CBF and CBP. However, as shown in the following samples (sample #3 and sample #4), ciliary function gradually improved, reaching or even exceeding baseline levels.

Furthermore, SNOT-22 scores aligned with the ciliary function results, showing a notable reduction from 45 in sample #1 to 13 in sample #4. This improvement corresponds to the switch to omalizumab treatment and restoration of ciliary function, strengthening the potential synergistic effect of functional sinus surgery and omalizumab in the management of CRSwNP.

In summary, despite attempts with oral corticosteroids and sinus surgery, CBF, CBP, and SNOT-22 remained altered in this patient. However, following omalizumab therapy, CBF and CBP were successfully normalized. SNOT-22 showed a notable improvement by 32 points. A direct relationship between improved CBP and CBF values and lower SNOT-22 scores was observed (i.e., 13/110 post-omalizumab), while poorer CBP and CBF values corresponded to higher SNOT-22 scores (i.e., 45/110 pre-omalizumab). Omalizumab achieved better control of ciliary function (CBF and CBP) and symptoms, while avoiding the known side effects of oral corticosteroids.

To date, no similar cases have been published showing ciliary function by DHSV across different treatment lines. A large-scale study could be interesting concerning the introduction of biotherapy such as omalizumab, in the monitoring of ciliary function. Previous studies carried out by DHSV mainly studied CBF after cell culture [1, 3, 14, 15]. The objective of this case was to analyze ciliary function on the same day of sampling while maintaining the conditions of inflammation to which the respiratory mucosa was chronically affected.

## Figures and Tables

**Table 1:** Results of ciliary function and SNOT-22 score across different treatment conditions in a patient presenting with CRSwNP.

	CBF (Hz)	CBP (percentage of normal beating)	SNOT-22
Sample #1 (preoperative)	16.78 ± 1.51	95.65	45
Sample #2 (postoperative)	14.25 ± 4.25	41.18	45
Sample #3 (1 month omalizumab)	13.60 ± 2.75	90	Not evaluated
Sample #4 (3 months omalizumab)	16.03 ± 1.72	100	13

The first sample was taken preoperatively, under oral corticosteroids. The second sample was taken postoperatively, under intranasal glucocorticosteroid. The third sample was taken after one month of omalizumab injections. The fourth sample was taken after 3 months of omalizumab injections. CBF (Hz) = ciliary beat frequency in hertz; CBP = ciliary beat pattern; SNOT-22 = 22-Item Sinonasal Outcome Test. Numerical values in CBF column are expressed as mean ± SD (standard deviation).

## Data Availability

The data supporting the findings of this case report are available upon request. Interested parties may obtain access to the data by contacting the authors via e-mail at Lioneljbenchimol@gmail.com. We are committed to promoting transparency and facilitating the sharing of research materials to contribute to the scientific community's understanding.
